# A clustering analysis of lipoprotein diameters in the metabolic syndrome

**DOI:** 10.1186/1476-511X-10-237

**Published:** 2011-12-19

**Authors:** Alexis C Frazier-Wood, Stephen Glasser, W Timothy Garvey, Edmond K Kabagambe, Ingrid B Borecki, Hemant K Tiwari, Michael Y Tsai, Paul N Hopkins, Jose M Ordovas, Donna K Arnett

**Affiliations:** 1 Department of Epidemiology, University of Alabama at Birmingham, School of Public Health, AL, USA; 2 Department of Biostatistics, Section on Statistical Genetics, University of Alabama at Birmingham, School of Public Health, AL, USA; 3 Department of Medicine, Division of Preventive Medicine, University of Alabama at Birmingham, AL, USA; 4 Birmingham VA Medical Center, Birmingham, AL, USA; 5 Nutrition Obesity Research Center, University of Alabama at Birmingham, School of Public Health, AL, USA; 6 Division of Statistical Genomics, Department of Genetics, Washington University, School of Medicine, 4444 Forest Park Boulevard-Box 8506, St. Louis, MO, USA; 7 Department of Laboratory Medicine and Pathology, University of Minnesota, MN; 8 Department of Internal Medicine, University of Utah, Salt Lake City, UT, USA; 9 JM-USDA-HNRCA, Tufts University, Boston, MA, USA; 10 The Department of Epidemiology and Population Genetics. Centro Nacional Investigación Cardiovasculares (CNIC) Madrid, Spain; 11 IMDEA Food, Madrid, Spain

**Keywords:** lipoprotein particle diameter, insulin resistance, nuclear resonance spectroscopy, Metabolic Syndrome, latent class analysis, GOLDN, waist circumference, hypertension, hypertriglyceridemia, fasting glucose

## Abstract

**Background:**

The presence of smaller low-density lipoproteins (LDL) has been associated with atherosclerosis risk, and the insulin resistance (IR) underlying the metabolic syndrome (MetS). In addition, some research has supported the association of very low-, low- and high-density lipoprotein (VLDL HDL) particle diameters with components of the metabolic syndrome (MetS), although this has been the focus of less research. We aimed to explore the relationship of VLDL, LDL and HDL diameters to MetS and its features, and by clustering individuals by their diameters of VLDL, LDL and HDL particles, to capture information across all three fractions of lipoprotein into a unified phenotype.

**Methods:**

We used nuclear magnetic resonance spectroscopy measurements on fasting plasma samples from a general population sample of 1,036 adults (mean ± SD, 48.8 ± 16.2 y of age). Using latent class analysis, the sample was grouped by the diameter of their fasting lipoproteins, and mixed effects models tested whether the distribution of MetS components varied across the groups.

**Results:**

Eight discrete groups were identified. Two groups (N = 251) were enriched with individuals meeting criteria for the MetS, and were characterized by the smallest LDL/HDL diameters. One of those two groups, one was additionally distinguished by large VLDL, and had significantly higher blood pressure, fasting glucose, triglycerides, and waist circumference (WC; *P *< .001). However, large VLDL, in the absence of small LDL and HDL particles, did not associate with MetS features. These associations held after additionally controlling for VLDL, LDL and HDL particle concentrations.

**Conclusions:**

While small LDL diameters remain associated with IR and the MetS, the occurrence of these in conjunction with a shift to overall larger VLDL diameter may identify those with the highest fasting glucose, TG and WC within the MetS. If replicated, the association of this phenotype with more severe IR-features indicated that it may contribute to identifying of those most at risk for incident type II diabetes and cardiometabolic disease.

## Introduction

IR is defined as cellular resistance to insulin stimulated glucose uptake. The compensatory hyperinsulinemia associates with a set of metabolic features that characterize the metabolic syndrome. The National Cholesterol Education Program (NCEP) Adult Treatment Panel (ATP) III defined the MetS as the presence of three or more of the following features: *1*) increased WC *2*) elevated TGs; *3*) low levels of HDL cholesterol (HDL-C); *4*) hypertension and, *5*) impaired fasting glucose (1). Given the atherogenic lipid profile seen in the MetS, and the additional hypertension and central obesity, it is not surprising that IR has been shown to be a major risk factor for cardiovascular diseases (CVD) [1,2)].

In addition to the traditional risk factors, an increased concentration of smaller LDL particles is considered a biological marker of atherosclerosis risk, IR [3-5] and the both the presence of the MetS [6,7] and its individual components, including raised TG [5,8] and fasting glucose [4,9], lowered HDL-C [8], increased WC [10] and hypertension [11]. In addition to the smaller LDL particles seen in MetS, some preliminary evidence suggest associations between IR and larger VLDL particles, and between IR and smaller HDL particles [3,4,12-14]. Despite these associations, how the diameters covary across the three fractions is poorly understood, and, as yet, information on all three fractions of lipoprotein diameter has not been captured into a single phenotype and examined for its relationship to disease.

Our first aim was to report how the diameters correlate across VLDL, LDL and HDL particles and confirm the association between the diameter of each fraction of lipoprotein and features of the MetS. Our second aim was to capture the relationship between all three fractions into a single trait, by clustering individuals into groups according to their similarities across all three fractions of lipoprotein simultaneously. We further aimed to examine the distribution of the MetS, and its individual components across these groupings, and assess whether any association between group and IR help when controlling for overall lipoprotein concentrations. This latter step was conducted to ascertain whether any association between MetS features and lipoprotein diameter occurs independently of lipoprotein concentrations, which has been the focus of much previous research.

## Results

### Group characteristics in lipoproteins, and their association with components fo the MetS

Table 1 gives sample characteristics for lipoprotein subfraction distribution. Within the sample as a whole, LDL diameter was highly correlated with HDL diameter (r = .78; *P *< 0.0001), but VLDL diameter was not significantly correlated with LDL diameter (r = -.02; *P *= 0.51) nor HDL diameter (r = .02; *P *= .54).

**Table 1:** Means (± standard deviations), or percentages, for lipoprotein, demographic and MetS characteristics across the GOLDN study population (N = 1036)

Characteristic	Mean (± standard deviations), or percentage
**Demographics**	
Male, %	47.8
Age, y	48.8 (16.2)
**MetS features**	
WC; cm	96. 7 (16.8)
Fasting glucose, (mmol/L)	5.63 (1.0)
Diabetes, %	8.2
Raised blood pressure (> 130/> 85 mm Hg), %	29.8
Systolic blood pressure, mm Hg	116.1 (16.7)
Diastolic blood pressure, mm Hg	68.6 (9.7)
Fasting TGs (mmol/L)	1.6 (1.3)
Fasting HDL-C (mmol/L)	1.2 (0.3)
Average no. of MetS counts per group member	2.2 (1.5)
MetS, %	38.2
**Sera lipoprotein particle concentrations (mmol/L)**	
VLDL	74.1 (49.8)
LDL	1374.1 (472.7)
HDL	31.1 (5.6)
**Average particle diameter (nm)**	
VLDL	51.3 (7.8)
LDL	20.8 (0.9)
HDL	8.8 (0.5)

Table 2 presents the correlations between individual particle diameters and components of the MetS. LDL and HDL particle diameters were significantly correlated with all MetS components (*P *< .001). VLDL correlated with MC, fasting glucose and TGs and systolic BP (P < .001), but did not correlate with HDL-C nor diastolic BP (*P *> .05).

**Table 2:** Pearson correlation coefficients between MetS features and lipoprotein diameters

	Lipoprotein Diameters		
**Variable**	**VLDL**	**LDL**	**HDL**

WC	0.14*	-0.41*	-0.43*
Fasting glucose	0.12*	-0.28*	-0.26*
Fasting TGs	0.15*	-0.61*	-0.50*
HDL-C	-0.01	0.69*	0.73*
Systolic BP	0.13*	-0.22*	-0.16*
Diastolic BP	0.05	-0.23*	-0.20

*p < 0.001;

### Eight groups of lipoprotein diameter clustering

LCA identified 8 groups (classes) of individuals. Group characteristics are reported in table 3. All groups showed a good internal reliability of α > 0.7. Group 7 consisted of only 7 individuals, while the sample size of the other groups ranged between 43-242 subjects, so data from group 7 should be interpreted with caution. However, analysis without group 7 did not change the pattern or significance of the results, and thus, the results are presented for the full data sample.

**Table 3:** Means (± standard deviations), or percentages, for characteristics of each particle diameter group

	1	2	3	4	5	6	7	8	p-value *
N	200	51	242	237	176	43	7	80	
α†	.76 (.16; .76-.80)	.80 (.18; .75-.85)	.74 (.18; .72-.76)	.72 (.16; .7-.74)	.74 (.16; .72-.76)	.78 (.17; .73-.83)	.998 (.01; .99-1.00)	.82 (.18; .78-.86)	
Male, %	72	61	60	44	28	19	43	14	< 0.0001
Age, y	50.5 (15.0)	50.9 (14.8)	50.6 (15.6)	46.8 (16.7)	47.5 (16.5)	41.1 (17.5)	53.8 (26.1)	49.7 (16.3)	0.001
**Average particle diameter (nm)**									
VLDL	53.1 (3.33)	65.7 (4.9)	45.6 (3.1)	53.6 (3.4)	45.3 (3.0)	64.2 (5.2)	98.1 (14.6)	50.4 (4.1)	< 0.0001
LDL	19.8 (.4)	20.1 (.6)	20.4 (.4)	21.1 (.4)	21.5 (.4)	21.7 (.6)	21.9 (.6)	22.2 (.4)	< 0.0001
HDL	8.4 (.2)	8.5 (.2)	8.6 (.2)	9.0 (.2)	9.2 (.2)	9.4 (.3)	9.7 (.3)	9.7 (.3)	< 0.0001
**Distributions of lipoprotein subfractions (sera concentrations; nmol/L)**									
*VLDL*									
Small	39.5 (25.6)	26.2 (17.3)	43.5 (23.8)	27.6 (14.3)	32.6 (18.2)	11.1 (8.7)	4.6 (7.0)	18.6 (14.2)	< 0.0001
Medium	61.2 (37.5)	45.6 (51.3)	44.4 (33.7)	27.8 (34.0)	28.1 (24.4)	8.9 (7.3)	3.0 (2.3)	17.7 (14.8)	< 0.0001
Large	7.88 (5.5)	17.8 (24.6)	1.8 (1.9)	3.8 (5.3)	.8 (1.0)	1.4 (2.3)	.4 (0.4)	.9 (1.3)	< 0.0001
*LDL*									
Very small	1208.4 (400.9)	1005.9 (359.3)	886.2 (374.8)	546.5 (266.4)	364.7 (227.4)	298.9 (273.0)	274.1 (219.1)	171.0 (156.8)	< 0.0001
Small	1516.6 (462.2)	1313.9 (466.4)	1178.7 (410.5)	745.2 (289.2)	528.1 (256.7)	431.0 (333.6)	351.0 (270.5)	215.4 (190.0)	< 0.0001
Medium small	308.2 (123.4)	308.1 (219.5)	292.5 (212.1)	198.6 (176.6)	163.4 (190.9)	132.1 (174.0)	76.89 (55.88)	44.7 (38.15)	< 0.0001
Large	154.6 (116.1)	238.5 (215.7)	291.3 (146.7)	470.8 (189.7)	560.7 (228.3)	630.1 (192.4)	775.99 (271.34)	746.86 (245.26)	< 0.0001
*HDL*									
Small	25.0 (5.0)	24.6 (5.4)	23.7 (4.9)	20.6 (4.7)	19.9 (5.1)	16.4 (4.9)	16.58 (4.74)	17.89 (4.51)	< 0.0001
Medium	1.5 (2.7)	2.7 (3.3)	1.9 (2.5)	4.3 (3.9)	4.0 (4.2)	5.8 (4.2)	3.22 (5.22)	2.43 (3.02)	< 0.0001
Large	3.0 (1.7)	3.3 (1.9)	4.6 (1.8)	6.9 (2.4)	8.7 (2.3)	10.0 (2.5)	11.50 (3.41)	12.42 (2.44)	< 0.0001
**Sera lipoprotein particle concentrations (nmol/L)**									
VLDL	108.6 (51.4)	76.4 (54.2)	80.4 (39.1)	58.5 (38.7)	61.2 (29.8)	28.8 (12.6)	11.60 (5.21)	42.19 (20.69)	< 0.0001
LDL	1760.0 (469.2)	1624.3 (488.8)	1520.8 (424.6)	1202.6 (353.6)	1086.4 (330.6)	1025.0 (338.5)	1050.72 (205.79)	969.10 (304.47)	< 0.0001
HDL	29.1 (5.5)	30.2 (5.3)	29.4 (5.7)	31.0 (5.5)	31.7 (5.1)	31.8 (5.5)	31.89 (6.44)	32.01 (5.19)	< 0.0001

*Controls for family structure; Values in the table are means+/-SD, etc.

† α is the internal reliability of the class and values above 0.70 are considered reliable [18].

#### Group characteristics in lipoprotein concentrations

Notably group 6 had the second largest VLDL diameter, but did not have a high concentration of VLDL particles, indicating that VLDL particle diameter gives information that is not analogous to the absolute concentration of the VLDL fraction. This was not the case for the LDL fraction, where the average diameter decreased as the LDL concentration increased.

#### Group characteristics in components of the MetS

LCA group differences were examined using mixed models that controlled for age and sex, as well as adjusting for between center differences and familial correlation. WC (*P *< 0.0001, Table 3), fasting blood glucose (*P *< 0.0001, Table 3), fasting TG (*P *< 0.0001, Table 3), fasting HDL-C concentrations (*P *< 0.0001, Table 3), the prevalence of diabetes (*P *< 0.0001, Table 3) and the percentage of group members with elevated BP (> 130/> 85 mm Hg; *P *= 0.002, Table 3) all differed significantly between the 8 groups.

#### Statistical adjustment for overall lipoprotein concentrations

All group differences remained significant (*P *< 0.0001) when models simultaneously controlled for fasting VLDL, LDL and HDL concentrations; except for fasting glucose which showed only a trend (*P *= 0.05; Table 3) for significance.

### Comparisons between groups meeting the NCEP ATP-III criteria for the MetS

Overall, groups 1 and 2 had the greatest number of individuals (~75%) that met the ATP-III criteria for the MetS. Groups 1-2 showed the smallest LDL diameters, yet the largest VLDL diameters were contained in groups 2, 6 and 7.

In *post hoc *analyses, groups 1 and 2 were not significantly different from each other with regard to the number of individuals who met the ATP III criteria for the MetS (*P *= 0.80), but WC, TG, glucose and diabetes prevalence were significantly higher (*P *< 0.001) in LCA group 2 compared to group 1. Although LDL and HDL diameter did not differ between groups 1 and 2 (*P *> .05), group 2 had significantly larger VLDL diameters (*P *< 0.0001) than group 1. As large VLDL diameter alone did not associate with MetS features, this indicates for the first time that it is the pattern of VLDL to LDL or HDL diameter that is indicative of MetS feature severity.

## Discussion

The aim of this study was examine how the diameters of three fractions of lipoprotein co-varied, and to create a phenotype that, for the first time, reflects the pattern of lipoprotein diameters across VLDL, LDL and HDL particles. By examining the co-variation of this phenotype with the individual components of the MetS, we report that the MetS occurs alongside a reduction in LDL and HDL particle diameters. However, although VLDL diameter alone does not associate with MetS features, some of the most extreme IR features (the highest glucose, diabetes prevalence, TG, and WC) occur alongside smaller LDL and HDL particles, simultaneously with larger VLDL particles. As increased fasting glucose and TGs and increased WC are important indicators of CVD risk, the pattern of VLDL-LDL (or VLDL-HDL) diameters may have implications for starting identifying the highest risk groups for progression of IR into type II diabetes and cardiovascular disease.

Although previous studies into the role of LDL particle size in the MetS have largely used an increase in the concentration of the small LDL subfraction(s) as a marker of the MetS, previous analyses using particle diameters have been shown to be analogous to results using the concentration of particle subfractions [4]. Using diameters as trait components, instead of subfraction concentrations, has two potential advantages: [1] there is no clear agreement on the number of subfractions within each fraction. Diameters are measured in nm and are a standardized unit. Using a standardized measurement (such NMR-based nm scale) over subfractions based on centrifugation and related techniques, makes replication in independent samples more straightforward, and may ease the use of information in clinical settings. The average diameter may reflect the distribution across NMR-based subfractions more than a single subfraction concentration alone. That is, an increase in a particular subfraction concentrations does not give information about whether this reflects an overall increase across all the subfractions. Overall shift in average particle diameter reflects subfraction distribution.

Our descriptive analysis reports that the MetS, and its individual features were characterized by small LDL and HDL particles. Previous research suggests that an increased concentration of small LDL particles is considered a marker of raised TG [5,8], raised fasting glucose [4,9], lowered HDL-C [8], increased WC [10] and hypertension [11]. Much of the research supports a shift to smaller HDL in IR [3,4,12-14]. The groups created during the LCA showed significant differences in all components of the MetS, and so our study unifies previous information, and supports the association of small LDL [7] and HDL particles with the components of the MetS.

To extend our understanding of how lipoprotein diameters may be a marker of IR features, LCA analysis grouped individuals by their similarities for each of the three fractions of lipoprotein. Thus, the groups reflected relationships between the diameters of the various fractions of lipoprotein within a unified trait. As LDL particle diameter increased across the groups, so did HDL particle diameter. VLDL diameter was not correlated with LDL diameter, which is important as it indicates at least partially separable genetic and/or environmental influences to LDL and VLDL particle diameter formation.

The groups containing individuals with small LDL and HDL diameters are highly enriched for the presence of the MetS, a risk factor for incident diabetes and cardiometabolic disease [1,2]. The inclusion of VLDL diameter into the particle diameter pattern stratified those with the MetS into two further groups, one of which (group 2) has more extreme IR features i.e., increased WC, glucose, TG and diabetes prevalence, although there was not a significant difference in the average number of MetS components between the groups. Thus, when LDL diameter is known, the inclusion of HDL diameter in a trait is minimally informative. But knowledge of the VLDL diameter increased trait sensitivity as to the degree of abnormality across several of the individual components of the MetS. Given the relationship of IR to and cardiovascular events [2], this trait may provide, or contribute to, a useful tool, available from a quick serum test, for identifying those at the highest risk of incident diabetes or cardiometabolic disease from those who meet MetS criteria.

To confirm that the associations between the groups and components of the MetS were not confounded by an association between particle diameter and overall particle numbers, we additionally controlled for lipoprotein concentrations. In these models, WC, fasting TG, systolic BP and HDL-C did differ significantly between the groups (*P *< 0.0001), but fasting glucose and diastolic BP showed only a trend towards an association (*P *= 0.05). Thus, the association between the mean value for components of the MetS and a trait created from fasting particle diameter pattern is not attributable to any shared association with overall lipoprotein concentrations.

Although the clustering of individuals by VLDL, LDL and HDL particle diameter may provide an important trait for future risk stratification, our study should be viewed in light of some limitations. Firstly, differences by race both in lipoprotein patterns, and in the association of lipoprotein pattern with MetS features needs examination, which was not possible within the current sample. Secondly, and crucially, the use of cross-sectional data precluded any causal inferences regarding predictive value of particle diameter clustering to the MetS, or regarding the association between particle diameter clustering and incident diabetes/cardiometabolic disorder. This is an important future direction for this work.

Overall, while LDL particle diameter correlated with HDL particle diameter (in a fasted state), VLDL diameter varied independently of LDL, and the inclusion of information on diameter from three fractions of lipoprotein was more informative about MetS features than that of a single fraction. The MetS was marked by small LDL and HDL diameters, but, together, large VLDL and small LDL and HDL diameters marked those with the highest glucose, diabetes prevalence, TG, and WC. Thus, together, a combination of LDL and VLDL diameter may provide the best tool for identifying those at increased risk for type II diabetes or CVD. It is of great interest to see, in longitudinal studies, if changes in lipoprotein diameter pattern occur before the presence of the MetS, or simply alongside, and to establish whether this pattern of small LDL and large VLDL particles can be shown to convey increased risk for incident diabetes and cardiovascular events.

## Material and methods

### Participants

The study population consisted of 1,328 men and women in the Genetics of Lipid-Lowering Drugs and Diet Network (GOLDN) study. All participants were white men and women recruited from Minneapolis, Minnesota and Salt Lake City, Utah. The primary aim of the GOLDN study was to characterize the role of genetic and dietary factors on an individual's response to fenofibrate; and, the details of the GOLDN study have been published elsewhere [15]. GOLDN consisted of an initial screening visit (visit 0) during which participants were asked to discontinue the use of lipid lowering drugs. Approximately 4 to 8 weeks later, baseline blood chemistries were measured (visit 1). A day later (visit 2) participants' blood samples were collected before (fasting) and after (postprandial) participating in a high fat meal challenge. On subsequent visits 3 and 4, fasting and postprandial blood samples were collected after a 3-week open label fenofibrate trial. For this analysis, we used fasting data from visit 2. This includes data only from subjects who were willing to participate in the high fat meal intervention. The final sample consisted of 1036 individuals across 187 families; 497 men and 539 women (mean ± SD: 48.8 ± 16.2 y of age). The protocol was approved by the Institutional Review Boards at the University of Minnesota, University of Utah, Tufts University/New England Medical Center and the University of Alabama at Birmingham. Written informed consent was obtained from all participants.

### Data collection

Clinical characteristics including anthropometric and blood-pressure measurements were taken at the study clinics where a fasting blood sample was also drawn, as described previously [15]. Questionnaires were administered to collect demographic data and information on lifestyle attributes and medical history.

### Anthropometric data

WC and BP data were collected by trained research staff who were instructed to take measurements against the skin or over lightweight non-constricting underwear. The tape was placed horizontally at the level of the umbilicus (navel), and the results recorded to the nearest centimeter, rounded down. BP data for both systolic and diastolic measurement were taken as the average of two consecutive readings which was rounded to the nearest integer.

### Biochemical measurements

All plasma samples used for this analysis were collected after an 8-hour fast and analyzed together at the end of the study. Measurements of overall plasma TG, VLDL, LDL, HDL and HDL-C concentrations were determined using enzymatic assays as previously described [16]. The serum concentrations of each subfraction are expressed in nmol/L. Measurements of VLDL, LDL and HDL diameter, and concentrations of each subfraction were determined by nuclear magnetic resonance (NMR) spectroscopy [17]. NMR detects the signal emitted by lipoprotein methyl-group protons when in the field of a magnet charged at 400 MHz. The NMR signal is deconvoluted to obtain estimates of particle numbers for each of several lipoprotein fractions. The weighted average particle diameter for each lipoprotein fraction (VLDL, LDL and HDL) is calculated as the sum of the average lipoprotein particle diameters multiplied by the relative mass percentage, based on the amplitude of the methyl NMR signal and given in nm. The ranges of diameters within each subfraction are shown in Table 4. Note that NMR groups IDL as a subclass of LDL [17].

**Table 4:** Diameter ranges of lipoprotein subclasses when measured by NMR [17]

NMR lipoprotein parameter	Diameter range (nm)
**VLDL**	
Large VLDL/chylomicrons	> 60
Medium VLDL	35-60
Small VLDL	27-35
**LDL**	
IDL	23-27
Large LDL	21.2-23
Small LDL	18-21.2
Medium small LDL	19.8-21.2
Very small LDL	18-19.8
**HDL**	
Large HDL	8.8-13
Medium HDL	8.2-8.8
Small HDL	7.3-8.2

### MetS components

The NCEP ATP III definition is used in this analysis, defined as the presence of three or more of the following features: *1*) WC > 102 cm [> 40 in] for men, > 88 cm [> 35 in] for women; *2*) TG ≥ 150 mg/dl; *3*) HDL-C; < 40 mg/dl in men, < 50 mg/dl in women; *4*) blood pressure (BP) ≥ 130 ≥ 85 mmHg; and *5*) impaired fasting glucose = > 100 mg/dl [1].

### Statistical analysis

Statistical analyses were carried out using SAS for Windows, version 9.2 (SAS Institute, Cary, NC).

### Initial analysis

Pearson correlations were run to examine the correlation between components of the MetS and lipoprotein diameters. For this analysis, TG concentrations and VLDL diameter were skewed and a natural logarithmic transformation applied to approximate a normal distribution.

### Clustering of individuals, into groups, based on similar VLDL, LDL and HDL diameters

Latent class analysis (LCA), a form of cluster analysis that groups individuals based on similarities within specified measures, was used to group study participants into 8 groups based on their average VLDL, LDL and HDL fractions. The aim of these analyses was to cluster together individuals who have a similar absolute average diameter for each of the three fractions of lipoprotein (VLDL, LDL and HDL).

### Advantages of LCA

Unlike commonly used difference methods, such as raw or residual difference scores, LCA can characterize means and differences across more than two measures. Further, LCA allows for a multinomial pattern of difference. Where responses can increase or decrease (e.g. LDL may be larger or smaller than VLDL), alternative methods can (1) oversimplify and (2) average out these complex difference patterns. LCA also allows a statistically significant assessment, through maximum-likelihood model fit comparisons, of the number of discrete groups within a population.

The LCA was implemented using the TRAJ Procedure in SAS [18]. LCA clusters individuals into classes according to similarities in the mean diameter for each fraction of lipoprotein using an iterative approach [19]. Initially, all participants are considered to be in a single class. Classes are added until an additional class does not improve the fit of the model as assessed using the log Bayes Factor (_log_*BF*). LCA uses maximum likelihood estimation, with standardized data to ease optimization.

**Figure 1:**
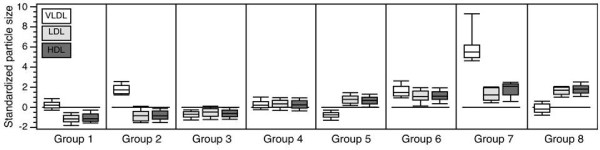
**Relative VLDL, LDL and HDL diameters by group**. Note: For display, the data are standardized within each fraction.

For each individual, the probability of membership of each group is given (which all sum to 1.00). Using a *maximum-probability assignment rule*, each individual is assigned to the group for which he/she has the highest probability of membership. The *average posterior probability *of group membership is the average of all the membership probabilities of all participants assigned to that group and is analogous to the internal reliability (the α) of the class. An average posterior probability of over 0.70 is considered reliable [19]. Figure 1 represents the results of the LCA, using standardized data for ease of display. For this figure only, each fraction of lipoprotein was standardized using the PROC STANDARD command.

#### Group characteristics

We used mixed models to determine whether the distribution of lipid particle subfraction concentrations and components of the MetS varied significantly by LCA groups. In these models, study center and pedigree were modeled as random effects.

Categorical MetS variables were modeled using PROC GENMOD while continuous ones were modeled using PROC MIXED in models that included LCA group, sex and age as fixed effects. The categorical MetS variables were hypertension and diabetes prevalence, while the continuous ones were WC, fasting glucose, TG and HDL-C concentrations. Systolic and diastolic measurements were included as continuous variables, for additional information.

Additional models were then run which controlled for VLDL, LDL and HDL concentrations.

#### Sensitivity analysis

To exclude the possibility that any significant between-group differences in components of the MetS were driven by a few extreme data points or outliers, all mixed models were additionally run without values +/- 4 SD from the mean of the outcome variable. The direction of results, and all significance levels remained the same, so results are reported here using the full sample.

## Abbreviations

BP: Blood pressure; HDL: High-density lipoprotein; IR: Insulin resistance; LCA: Latent class analysis; LDL: Low-density lipoprotein; MetS: Metabolic syndrome; NMR: Nuclear resonance spectroscopy; VLDL: Very-low density lipoprotein; WC: Waist circumference

## Competing interests

The authors declare that they have no competing interests.

## Authors' contributions

ACFW carried out the analysis and wrote the manuscript, SG, WTG, EKK, IBB, HKT, MYT, PNH and JMO helped with critical revision of the manuscript, DKA is PI of the study and helped with critical revision of the manuscript. All authors read and approved the final manuscript.
